# A nomogram to predict outcomes of lung cancer patients after pneumonectomy based on 47 indicators

**DOI:** 10.1002/cam4.2805

**Published:** 2020-01-03

**Authors:** Bo Cheng, Cong Wang, Bing Zou, Di Huang, Jinming Yu, Yufeng Cheng, Xue Meng

**Affiliations:** ^1^ Department of Radiation Oncology Cancer Hospital of Shandong Province Jinan P. R. China; ^2^ Department of Radiation Oncology Qilu Hospital Shandong University Jinan P. R. China

**Keywords:** hematological biomarkers, lung caner, nomogram, prognosis

## Abstract

**Aims:**

We aimed to establish a nomogram for lung cancer using patients' characteristics and potential hematological biomarkers.

**Methods:**

Principle component analysis was used to reduce the dimensions of the data, and each component was transformed into categorical variables based on cutoff values obtained using the X‐tile software. Multivariate analysis was used to determine potential prognostic biomarkers. Five components were used in the predictive nomogram. Internal validation of the model was performed by bootstrapping of samples, while external validation was performed on a separate cohort from Shandong Cancer Hospital. The predictive accuracy of the model was measured by concordance index and risk group stratification. Decision curve analysis was performed to evaluate the net benefit of the models.

**Results:**

One hundred patients in the Discovery group and 111 patients in the Validation group were retrospectively analyzed in this study. Forty‐seven indexes were sorted into eight subgroups. Five components based on cox regression analysis were enrolled into the predictive nomogram. The nomogram prediction of the probability of 3‐ and 5‐year overall survival was in great concordance with the actual observations. Of interest, the nomogram allowed better risk stratification of patients and better accuracy in predicting patients' survival compared with pathological tumor‐node‐metastasis staging system.

**Conclusion:**

A nomogram was established for prognosis of lung cancer, which can be used for treatment selection and clinical care management.

## Graphical abstract

We established a nomogram for survival prediction of lung cancer, which can be used for treatment therapy selection and clinical care option. The nomogram allowed better distinction between survival curves compared with pathological tumor‐node‐metastasis staging system.

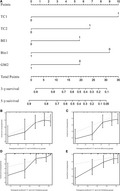

## 1 BACKGROUND

Lung cancer is the most lethal cancer worldwide. Despite the rapid advancement in diagnosis and treatment technologies in last decade, the prognosis of lung cancer remains poor.^1^ Tumor‐derived factors have been the main focus for establishing prognostic and predictive biomarkers of lung cancer. However, reliance on tumor tissue does not allow serial sampling of tumors over the treatment course, and does not allow multiple biopsy attempts for intra‐tumor heterogeneity assessment. Therefore, the identification of novel blood biomarkers to identify tumor tissues and to predict tumor behavior and patients' survival would greatly improve clinical practice. Indeed, blood‐based biomarkers can capture the molecular diversity of the disease, while the ease of serial testing enables the monitoring of its spatial and temporal progression.^2^

Accumulating studies have employed clinical factors such as hypertension history, diabetes history,^3^ and hematological biomarkers including hemoglobin (Hb), platelets (PLTs) and white blood cells (WBCs), in the prognostic analysis of lung cancer patients.^4^ However, these studies did not reach consensus on cutoff values and biomarkers selection, which may lead to inaccurate conclusions. For instance, a meta‐analysis reviewed 12 studies focusing on the prognostic value of PLTs in lung cancer patients.^5^ The cutoff values ranged between 300 × 10^9^/L and 450 × 10^9^/L, and the proportion of patients with elevated PLTs ranged between 6.9% and 58.5%. In addition, several studies have analyzed PLT count and PLT‐to‐lymphocyte ratio^6^ or PLT volume/PLT count ratio^7^ to investigate the prognostic significance of the combined index in lung cancer patients. The heterogeneity of the data leads to inconsistent conclusions among these studies. In this regard, principle component analysis makes it possible to identify potentially interpretable patterns in the data by weighting variables over a principal component. The resulting linear combination of the variables, weighted by their contribution to explain the variance in a particular orthogonal dimension, captures the variation among the study subjects in a highly interpretable manner.^8^

Nomogram is a commonly used tool to evaluate the prognostic biomarkers in oncology and medicine. With their ability to generate an individual numerical probability of a clinical event by integrating diverse prognostic and clinical variables, nomograms allow the development of potential biologically and clinically integrated models that bring us steps closer toward personalized medicine.^9^ Indeed, nomograms have been shown to be a better prediction tool in several types of cancer, compared with the traditional pathological tumor‐node‐metastasis (pTNM) staging system.^10^, ^11^ Despite that several nomograms have been generated for lung cancer, these nomograms did not include comprehensive biomarkers, and the outcome indicators were relapse or metastasis rates rather than the survival rate.^12^, ^13^, ^14^, ^15^ Therefore, we aimed to identify potential prognostic clinical and circulating biomarker indexes of lung cancer, which were used to develop a nomogram for survival prediction in a well‐defined Chinese cohort of lung cancer patients receiving pneumonectomy.

## 2 MATERIAL AND METHODS

### 2.1 Patients

A total of 100 lung cancer patients receiving pneumonectomy at Qilu Hospital between 2012 January and December of Shandong University were assigned as the Discovery group. A separate cohort of 111 patients at Shandong Cancer Hospital between 2013 January and 2014 January was assigned as the Validation group. Patient characteristics were collected including gender, age, smoking and drinking habits, high blood pressure and diabetes history, and family history of lung cancer. Tumor characteristics (TC) included size, site, pathology type, lymph node ratio, differentiation, and pTNM stage. In addition, blood measurements of patients before surgery were also collected, including: (a) routine blood biomarkers such as Hb and blood count of red blood cells, neutrophils, lymphocytes, monocytes, eosinophils, and basophils; (b) coagulation indicators such as PLTs, prothrombin time, international normalized ratio, activated partial thromboplastin time, fibrinogen and thrombin time; (c) glucolipid metabolism (GM) indicators such as cholesterol, triglyceride, high‐density lipoprotein (HDL), low‐density lipoprotein (LDL), and glucose; (d) liver function (LF) indicators including glutamic‐oxalacetic transaminase, glutamic‐pyruvic transaminase, alkaline phosphatase, total protein, albumin, globulin, and albumin‐to‐globulin ratio; (e) biochemical and electrolyte indicators such as K, Na, Ca, lactate dehydrogenase (LDH), and carbon dioxide combining power; and (f) renal function (RF) indicators including uric acid, creatinine, and blood urea nitrogen.

Patients were followed up every 6 months, and the inclusion criteria of patients were as follow: (a) patients who received pneumonectomy; (b) patients who were pathologically diagnosed and confirmed as lung cancer; (c) patients with complete follow‐up data. Patients were excluded if they had received neoadjuvant radiotherapy and chemotherapy, or if they refused to attend the study. Relapse, metastasis, and death time were recorded. This study was approved by the Ethics Committees of Shandong Cancer Hospital and Qilu Hospital. All patients included in this study provided signed informed consents.

### 2.2 Data assessment

Mean substitution method was used to impute missing data. The method replaces missing values with an average value of non‐missing elements of the corresponding variable^16^ (Table S1). All indexes were classified according to their clinical significance. Principle component analysis was used to reduce the dimensions of the data,^8^ and principle components with eigenvalues >1 were extracted. Each component was transformed into a binary variable according to optimal cutoff values that were defined based on the minimal *P* value approach using the X‐tile software (http://www.tissuearray.org/rimmlab).^17^ All components were subjected for univariate survival analysis using the Kaplan‐Meier method, and those with log‐rank *P* ≤ .1 were included in the multivariate Cox regression. The significant variables from the multivariate analysis were included into a backward step‐down process with an Akaike information criterion to build the nomogram. The nomogram was evaluated using 1000 bootstraps resampling of patients in the Discovery and the Validation groups. Calibration of the nomogram for 3‐ and 5‐year overall survival (OS) was performed by comparing the predicted survival with the observed survival after bias correction. The predictive performance of the model was evaluated using the receiver operating characteristic (ROC) curve together with concordance index (C‐index). In addition, we conducted a group‐stratified analysis of the total risk score to compare the discriminative ability of the nomogram with that of the pTNM staging system in the Discovery and the Validation cohorts. Decision curve analysis (DCA) was performed to evaluate the net benefit of the models. Statistical analysis was performed using the IBM SPSS statistics version 24.0 (SPSS) and using the *rms* and *Hmisc* statistical packages in R version 3.1.2 (http://www.r-project.org).^18^

## 3 RESULTS

### 3.1 Patients' characteristics

A total of 211 patients, including 145 males and 66 females, were retrospectively analyzed in this study (Table 1). A statistical summary of all measurements in the Discovery and the Validation groups is shown in Table S1.

**Table 1:** Characteristics of patients in discovery and validation groups

Characteristics	Discovery group (n = 100)		Validation group (n = 111)	
	N	%	N	%
Gender				
Male	69	69.0	76	68.5
Female	31	31.0	35	31.5
Age				
<60	45	45.0	54	48.6
≥60	55	55.0	57	51.4
Smoke index				
<400	54	54.0	71	64.0
≥400	46	46.0	40	36.0
Drink				
No	62	62.0	70	63.0
Yes	38	38.0	41	37.0
HBP				
No	80	80.0	85	76.6
Yes	20	20.0	26	23.4
Diabetes				
No	97	97.0	102	91.9
Yes	3	3.0	9	8.1
Tumor site				
Left upper lobe	25	25.0	32	28.8
Left lower lobe	25	25.0	21	18.9
Right upper lobe	26	26.0	30	27.0
Right middle lobe	8	8.0	6	5.4
Right lower lobe	16	16.0	22	19.8
Tumor size				
<3 cm	41	41.0	63	56.8
≥3 cm	59	59.0	48	43.2
T stage				
T1	43	43.0	32	28.8
T2	45	45.0	67	60.4
T3	11	11.0	11	9.9
T4	1	1.0	1	0.9
N stage				
N0	56	56.0	76	68.5
N1	38	38.0	13	11.7
N2	6	6.0	21	18.9
N3	0	0.0	1	0.9
pTNM stage				
I	44	44.0	71	64.0
II	42	42.0	16	14.4
III	14	14.0	24	21.6
IV	0	0.0	0	0.0
Differentiation				
Poor	39	39.0	40	36.0
Middle	51	51.0	46	41.4
High	8	8.0	25	22.5
Undifferentiated	2	2.0	5	10.4
Pathology type				
Squamous carcinoma	43	43.0	33	29.7
Adenocarcinoma	52	52.0	68	61.3
Small cell carcinoma	1	1.0	1	0.9
Other	4	4.0	9	8.1
Treatment				
Radiotherapy	4	4.0	1	0.9
Chemotherapy	21	21.0	53	47.7
Chemoradiotherapy	12	12.0	7	6.3
None	63	63.0	50	45.0

Abbreviations: HBP, high blood pressure; pTNM, pathological tumor‐node‐metastasis.

### 3.2 Principle component analysis

**Figure 1:**
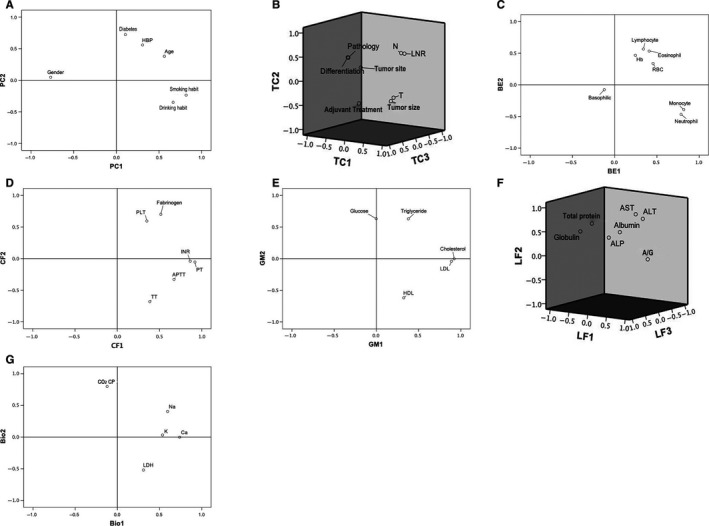
The component scatter plots showing the degree of variable variation explained by each component. The component scatter plots for PC(A), TC(B), BE(C), CF(D), GM(E), LF(F) and Bio(G) were descripted. Abbreviations: AGratio, albumin‐to‐globulin ratio; ALP, alkaline phosphatase; ALT, glutamic‐pyruvic transaminase; APTT, activated partial thromboplastin time; AST, glutamic‐oxalacetic transaminase; BE, blood routine examination; Bio, biochemical biomarkers; CF, coagulation function; GM, glucolipid metabolism; Hb, hemoglobin; INR, international normalized ratio; LF, liver function; LDH, lactate dehydrogenase; LNR, lymph node ratio; PC, patients’ characteristics; PLT, platelet; PT, prothrombin time; RBC, red blood cell; N, N stage; T, T stage; TC, tumor characteristic; TT, fibrinogen, thrombin time

Up to 47 indexes were collected, including patients' clinical characteristics and blood examination (BE) results. All the indexes were sorted according to their clinical significance into the following eight categories: patients' characteristics (PC), TC, routine BE measurements, coagulation function indicators (CF), GM, LF indicators, biochemicals and electrolytes (Bio), and RF indicators. Principle component analysis was applied on the collected indexes and 18 principle components with eigenvalues >1 were extracted for survival analysis, two of which were from the PC category, four from TC, two from BE, two from CF, two from GM, three from LF, two from Bio, and one from RF category. The component scatter plot in Figure 1 shows the degree of variable variation explained by each component. The component score coefficient matrix is displayed in Table S2. Each component in the Validation group was then calculated according to the eigenvalues and the component score coefficients. The formulas are shown in Formula S1. Each component was then transformed into a binary variable based on certain cutoff values that were calculated using the X‐tile software (Table S3).

### 3.3 Survival analysis

The survival time ranged between 4 and 80 months in the Discovery group, with a median survival time of 60 months. Univariate OS analysis indicated the significance of TC1 (*P* = .001), TC2 (*P* = .068), BE1 (*P* = .014), BE2 (*P* = .003), CF2 (*P* = .010), GM1 (*P* = .011), GM2 (*P* = .069), LF1 (*P* = .021), LF3 (*P* = .073), Bio1 (*P* = .017), and RF (*P* = .065) in 5‐year survival prediction. These indexes were included in a multivariate analysis, which identified the following indexes as potential biomarkers for 5‐year OS prediction: TC1 (*P* = .012, HR = 3.1, 95% confidence interval (CI) [1.280‐7.510]), TC2 (*P* = .005, HR = 2.796, 95% CI [1.363‐5.738]), BE2 (*P* = .048, HR = 0.376, 95% CI [0.143‐0.990]), GM2 (*P* = .031, HR = 0.471, 95% CI [0.238‐0.933]), and Bio1 (*P* = .020, HR = 0.402, 95% CI [0.187‐0.865]) (Table 2).

**Table 2:** Univariate and multivariate analysis results in Discovery group

Components	Univariate analysis	Multivariate analysis		
	*P* value	HR	95% CI	*P* value
PC1	.549			
PC2	.538			
TC1	.001	3.100	1.280‐7.510	.012
TC2	.068	2.796	1.363‐5.738	.005
TC3	.111			
TC4	.134			
BE1	.014			
BE2	.003	0.376	0.143‐0.990	.048
CF1	.199			
CF2	.010			
GM1	.011			
GM2	.069	0.471	0.238‐0.933	.031
LF1	.021			
LF2	.145			
LF3	.073			
Bio1	.017	0.402	0.187‐0.865	.020
Bio2	.200			
RF	.065			

### 3.4 Development of survival prediction nomogram

**Figure 2:**
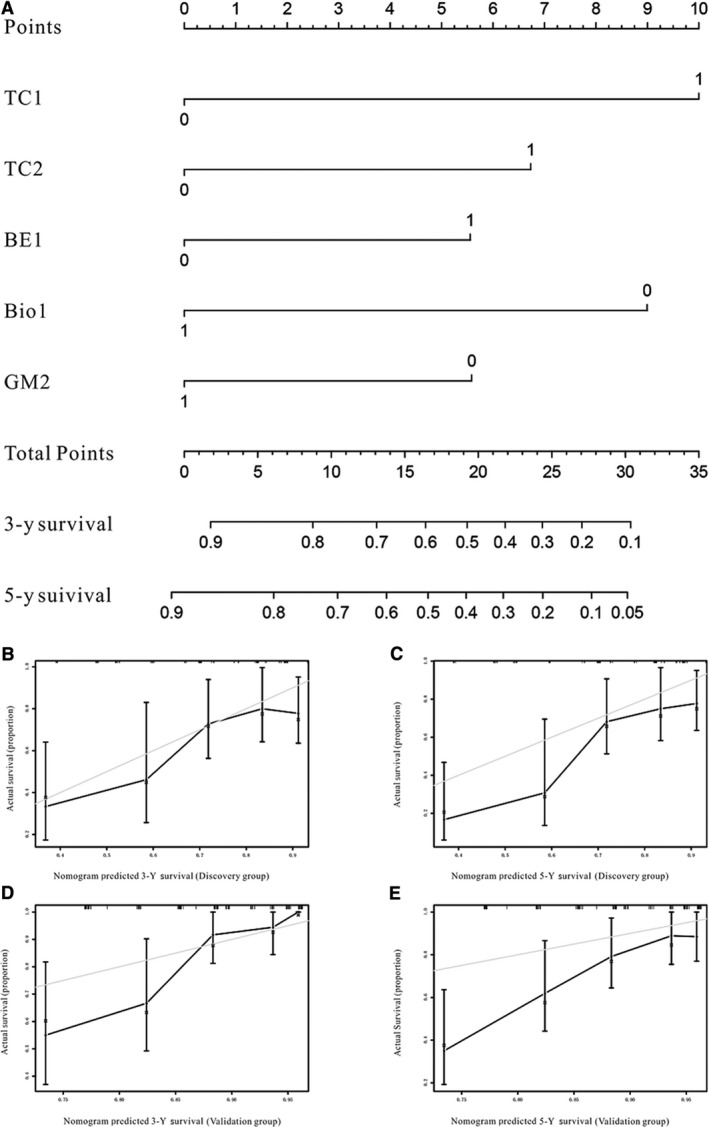
Nomogram predicting 3‐ and 5‐ survival after pneumonectomy for lung cancer patients (A). The calibration curves for predicting patient survival at (B, D) 3‐y and (C, E) 5‐y in the Discovery and the Validation groups. Nomogram‐predicted survival is plotted on the *x*‐axis; actual survival is plotted on the *y*‐axis. Abbreviations: BE, blood routine examination; Bio, biochemical biomarkers; GM, glucolipid metabolism; TC, tumor characteristic

The five independently associated index categories TC1, TC2, BE2, GM2, and Bio1 were used to establish the OS estimation nomogram. Bootstrap resampling was used for nomogram validation. The nomogram is shown in Figure 2, and the scoring system based on these five index categories is shown in Table 3. The nomogram showed that the TC1 category had the largest contribution to prognosis, followed by Bio1, TC2, GM2, and BE1. Each component was calculated and assigned as 0 if it was less than the cutoff value or as 1 if greater than the cutoff value. Each variable was then given a score on the point scale, and the total score of each component was calculated and identified on the total point scale, which allowed the calculation of the estimated 3‐ and 5‐year survival probabilities.

**Table 3:** Point assignment of each component and prognostic score for lung cancer patients

Group	Scores	Estimated 3‐y OS (%)	Estimated 5‐y OS (%)
TC1			
<−0.08	0		
≥−0.08	10		
TC2			
<0.63	0		
≥0.63	7		
BE1			
<0.64	0		
≥0.64	6		
GM2			
<−0.38	6		
≥−0.38	0		
Bio1			
<−0.78	9		
≥−0.78	0		
Total prognostic score^a^			
	2	90	
	9	80	
	13	70	
	16	60	
	19	50	
	22	40	
	24	30	
	27	20	
	30	10	
	0		90
	6		80
	10		70
	14		60
	17		50
	19		40
	22		30
	24		20
	28		10
	30		5

Abbreviation: OS, overall survival.

a For total prognostic score estimation, please refer to Figure 2.

### 3.5 Performance of the new scoring system

**Figure 3:**
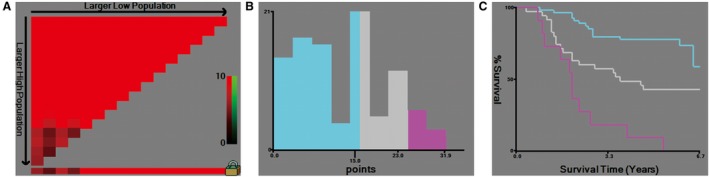
X‐tile analysis of survival based on Nomogram points. X‐tile plots of training group are shown in the left panel (A). The cut‐point in the left panel is shown on a histogram of the entire cohort (B), and a Kaplan‐Meier plot (C). (low points = blue, middle points = gray, high points = magenta)

**Figure 4:**
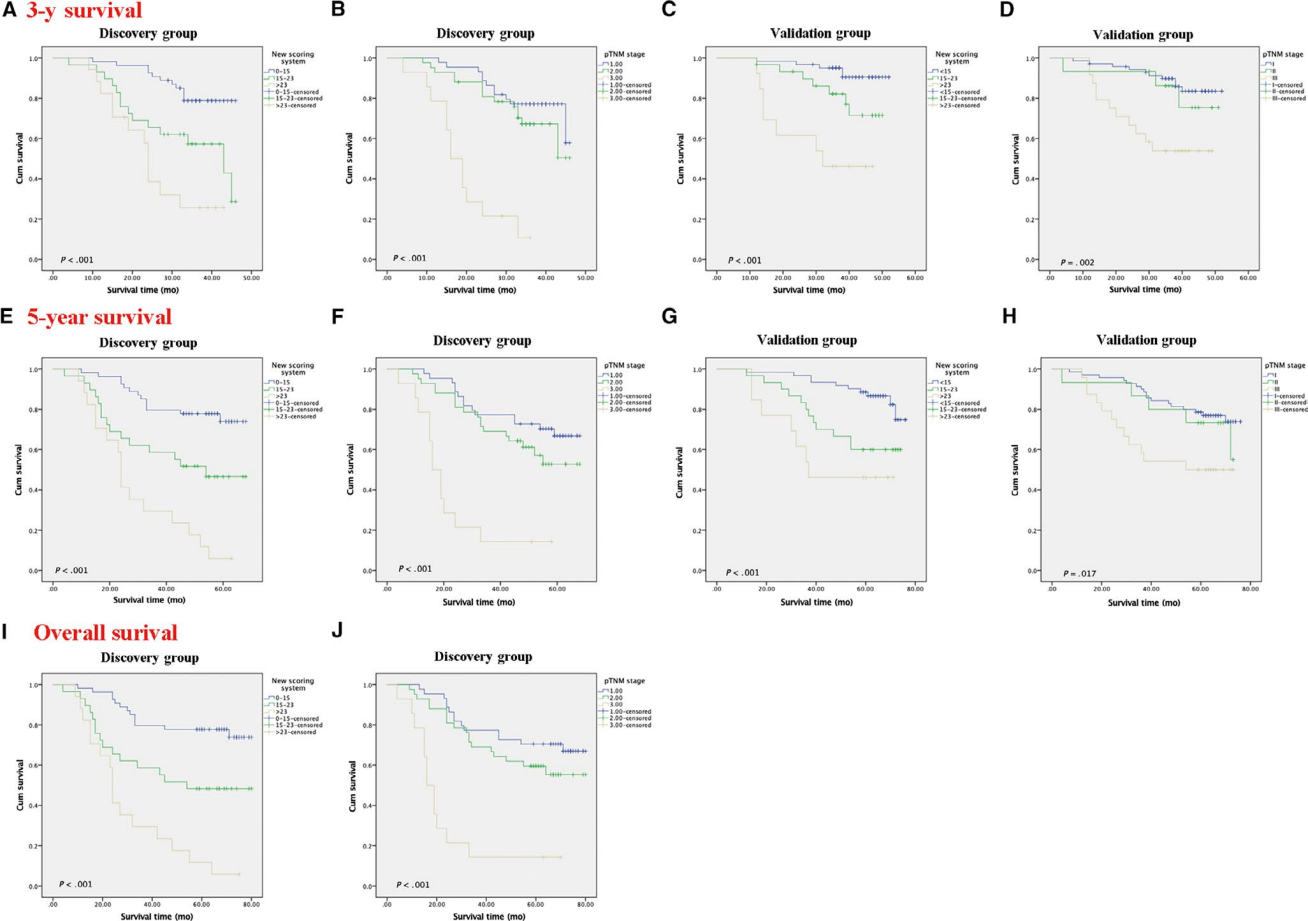
Kaplan‐Meier survival curves for patients with lung cancer predicted by new scoring system and pathological tumor‐node‐metastasis (pTNM) stage in the Discovery and Validation groups. The Kaplan‐Meier survival curves to predict 3‐y survival (A, C), 5‐year survival(E, G) and overall survival(I) in Discovery Group and Validation Group by the New scoring system as well as the Kaplan‐Meier survival curves to predict 3‐y survival (B, D), 5‐y survival (F, H) and overall survival(J) in Discovery Group and Validation Group by pTNM stage were shown

**Figure 5:**
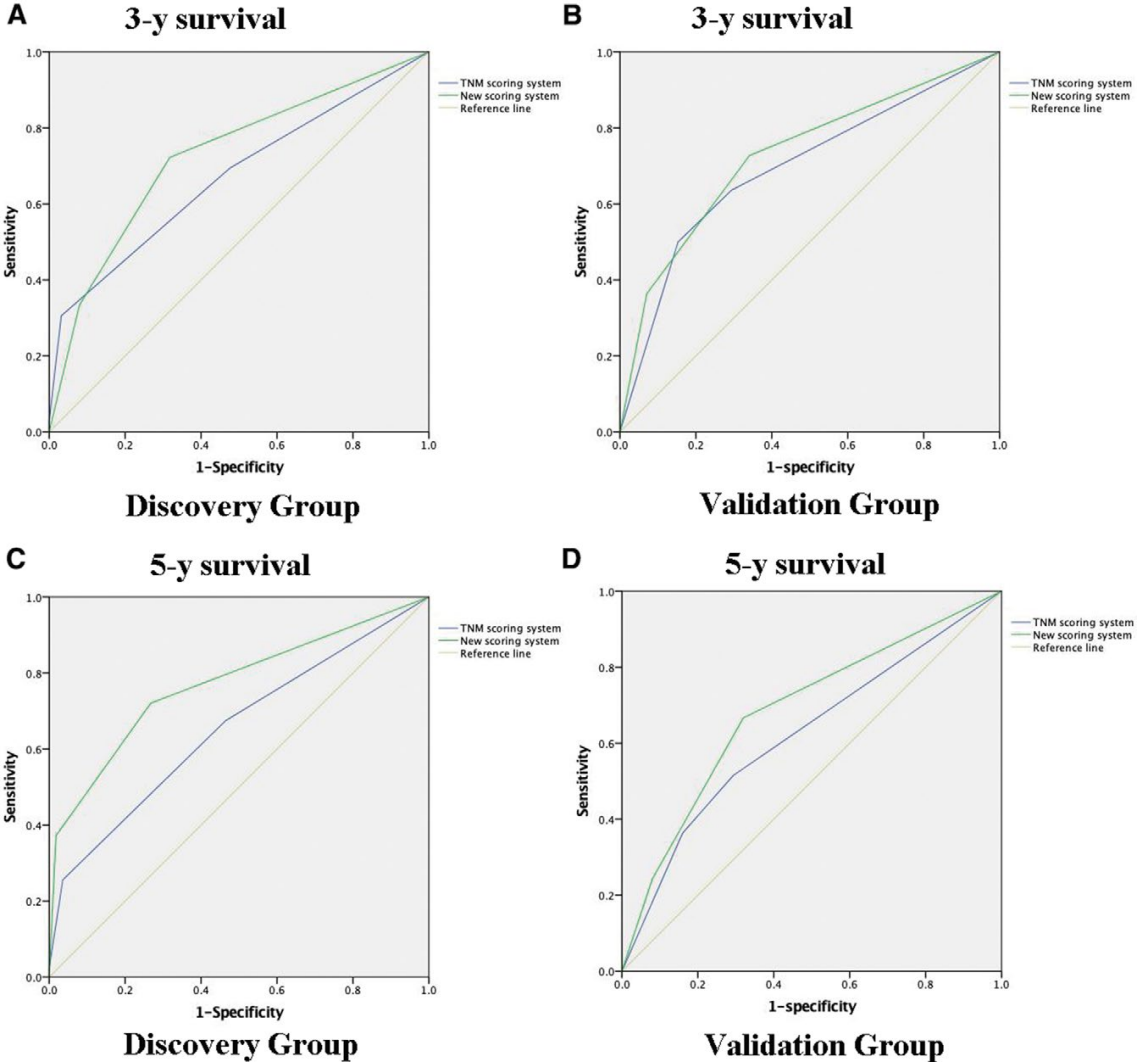
Receiver operating characteristic curve analysis for the sensitivity and specificity of the new scoring system and pathological tumor‐node‐metastasis (pTNM) scoring system to predict 3‐y survival(A, B) and 5‐y survival(C, D) in Discovery and Validation groups. New scoring system had higher accuracy compared with pTNM scoring system

The performance of the nomogram was graphically evaluated using a calibration curve. The calibration plots of the observed vs nomogram‐predicted 3‐ and 5‐year OS probabilities showed a strong agreement in the Discovery cohort and an acceptable agreement in the Validation cohort (Figure 2C‐F). The cutoff values for the total scores were classified according to the optimal cutoff analysis of the Discovery cohort into the following categories: <15, 15‐23, and >23 (Figure 3). Accordingly, we built the new scoring system to predict the survival of patients. We found that the new scoring system accurately predicted the 3‐ and 5‐year survival probabilities of patients in the Discovery group as well as in the Validation group (Figure 4A‐H). Patients were followed up up to 80 months in Discovery group, thus we also built the K‐M curve for overall survival. New scoring system also performed better than pTNM scoring system (Figure 4I,J). We also used ROC curve as well as C‐index to compare the performance (Table 4; Figure 5). The nomogram demonstrated a better accuracy in the estimation of 3‐ and 5‐year OS probability in Discovery group, compared with the pTNM staging system.

**Table 4:** C‐indexes for new scoring system and TNM staging system

	Staging systems	Discovery group			Validation group		
		C‐index	95% CI	*P*	C‐index	95% CI	*P*
3‐y OS prediction	New scoring system	0.727	0.620‐0.833	<.001	0.729	0.603‐0.856	.001
	TNM Staging system	0.671	0.556‐0.787	.005	0.696	0.563‐0.829	.005
5‐y OS prediction	New scoring system	0.770	0.672‐0.867	<.001	0.685	0.574‐0.797	.002
	TNM Staging system	0.653	0.542‐0.764	.009	0.623	0.504‐0.742	.042

Abbreviations: OS, overall survival; TNM, tumor‐node‐metastasis.

**Figure 6:**
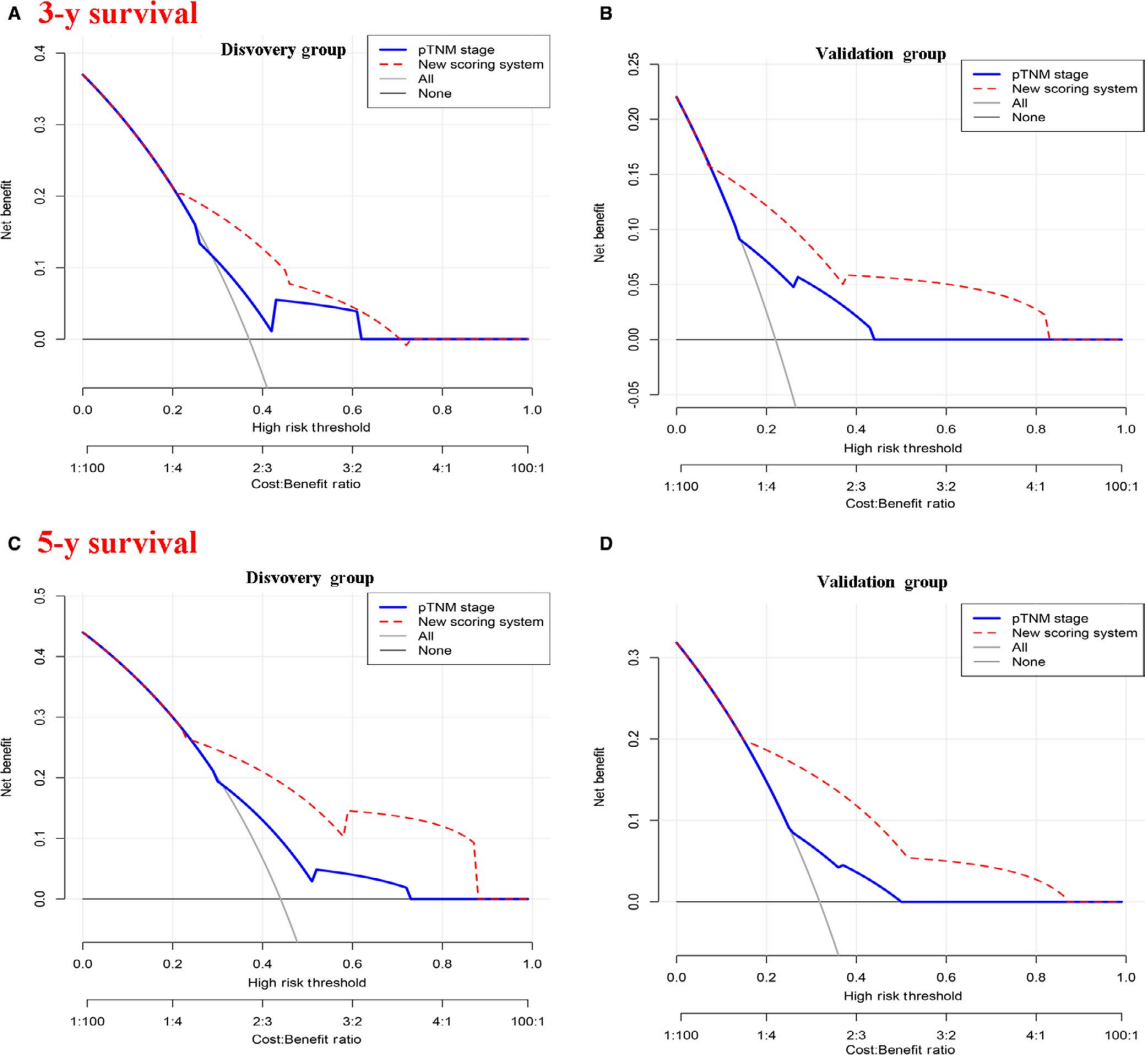
Decision curve analysis for the clinical benefit of the new scoring system and pathological tumor‐node‐metastasis (pTNM) scoring system. New scoring system behaved better than pTNM scoring system

Finally, to determine whether the predictive nomogram was clinically useful, DCA was performed to evaluate the net benefit of the models. Based on a continuum of potential thresholds for death (*x* axis) and the net benefit of using the model to risk‐stratify patients (*y* axis) relative to predict the 3‐ and 5‐year survival, the DCA graphically presented that the new scoring system was better than traditional pTNM system (Figure 6). Hence, this nomogram is the best model for predicting lung cancer patient survival, which might help clinicians with patient counseling, decision‐making, and follow‐up scheduling.

## 4 DISCUSSION

In the era of precision medicine, it is vital to include as much prognostic and predictive information as possible for decision‐making. The pTNM staging system remains the gold standard for the prognostic prediction of lung cancer. However, the pTNM system is unable to incorporate tumor, lymph nodes, and metastasis as continuous variables. Given the limitations of the pTNM staging system, the nomogram has emerged as a simpler and more advanced tool with numerous advantages.^9^ Several nomograms for lung cancer have been reported; however, they only included the clinical characteristics without considering circulating blood indicators. In contrast, some nomograms were built to predict brain metastasis and clinical targeted treatment outcomes.^13^, ^14^, ^19^ Several studies focusing on the significance of circulating blood markers showed that Hb, WBCs, PLTs, HDL, LDL, and tumor biomarkers could be promising prognostic biomarkers for lung cancer. Similarly, lipid metabolism was shown to be highly altered in lung cancer cells,^20^ and several studies have reported that low serum HDL,^21^ high LDH,^22^ and decreased total cholesterol^23^ are associated with a higher incidence of lung cancer. In addition, despite certain disagreements,^27^ patient's diabetes history has been associated with poor OS of lung cancer patients.^24^, ^25^, ^26^ Moreover, inflammation can substantially contribute to the development of malignancies by promoting tumor angiogenesis, metastasis, and proliferation, as well as by interfering with the response to systemic treatments.^28^, ^29^ For example, neutrophils and lymphocytes play vital roles in tumor inflammation, and an imbalance between neutrophils and lymphocytes ratio could lead to anti‐apoptotic effects and is considered a prognostic factor in lung cancer patients.^30^ Besides, HB concentration was reported to be an independent prognostic factor for OS and RFS in non‐small cell lung cancer.^31^ Serum albumin, on the other hand, can be useful for the identification of nutritional risk and postoperative complications,^32^ and higher serum albumin level was reported to be associated with better survival in lung cancer patients.^33^ Furthermore, RF and LF can affect the treatment options and outcomes of lung cancer patients.^34^ Therefore, by building up on these promising findings, we aimed to develop a nomogram for the prognosis of lung cancer patients by including all possible categories of cancer indicators, such as PC, tumor characteristics, and circulating biomarkers.

External validation is an important approach to determine the generalizability of developed nomograms. Therefore, we used the external data from another hospital to verify the model. Calibration plots showed optimal consistency between the predicted and the observed survival probabilities in the Discovery group, which was slightly reduced in the Validation cohort, especially for 5‐year survival prediction.

The discrimination between the new scoring system and the pTNM staging system was further revealed by the concordance measurement. The value of the C‐index ranges from 0.5 to 1.0, with 0.5 indicating a random chance and 1.0 indicating a perfect ability of the model for outcome prediction. The C‐index of the new scoring system was higher than the pTNM staging system in the Discovery group for 3‐year (0.727 vs 0.671, respectively) and 5‐year OS prediction (0.770 vs 0.653, respectively). In the Validation group, the discriminative ability of the new scoring system remained superior to that of the pTNM staging system for 3‐year OS prediction (0.729 vs 0.696, respectively) and 5‐year OS prediction (0.685 vs 0.623, respectively). Three risk groups were better stratified by the new scoring system in both the Discovery and the Validation cohorts, compared with the pTNM staging system. And the DCA graphically presented that the new scoring system was better than traditional pTNM system.

Missing data are a common problem in clinical trials and are often inadequately handled in the statistical analysis even in the top tier medical journals.^35^, ^36^ A majority of researchers exclude these cases from the data, which results in biased outcomes and a drop in the statistical power.^37^ In our study, we used the mean substitution, which replaces the missing values with an average value of non‐missing elements in the corresponding variable. This imputation method is very convenient to reconstruct the missing data instead of excluding incomplete cases from the study.

To the best of our knowledge, this is the first prognostic nomogram for lung cancer that considers patients' clinical characteristics in addition to circulating blood biomarkers. We believe that this nomogram provides comprehensive information for patients, which could provide a better guidance for clinical therapy. Based on this tool, potential higher risk patients with poor survival could be more precisely selected for a specific treatment strategy.

Finally, this study has some limitations that need to be addressed. First, some important indicators which have been reported to be significant in survival prediction, such as D‐dimer, C‐reactive protein, and circulating tumor biomarkers, were not included in this study due to missing data in our database. Second, this tool is limited by small population and the retrospective nature of the data collected.

In conclusion, we have developed a promising nomogram for predicting survival in lung cancer patients after esophagectomy. The nomogram is based on both clinical and circulating biomarkers, and provides a strong prognostic superiority over pTNM staging system in lung cancer patients. The nomogram can help clinicians to make better predictions of patient survival and to give improved individualized treatment recommendations for lung cancer treatment.

## CONFLICT OF INTEREST

None declared.
