# Supplementary material for: A nomogram to predict outcomes of lung cancer patients after pneumonectomy based on 47 indicators
Source: Cancer Med. 2020 Jan 3;9(4):1430–40. doi: 10.1002/cam4.2805 (PMC7013057; doi:10.1002/cam4.2805)
Supplement: Supplementary file 1 [file CAM4-9-1430-s001.docx]

|  | Discovery Group | | Validation Group | | |
| --- | --- | --- | --- | --- | --- |
|  | Mean | SD | Mean | SD |  |
| Age (years) | 60.25 | 9.23 | 59.61 | 10.72 |  |
| Hb (g/L) | 151.30 | 141.47 | 141.53 | 14.942 |  |
| RBC (10^12) | 4.35 | 0.44 | 4.57 | 0.55 |  |
| Neutrophil (10^9) | 4.60 | 2.30 | 4.08 | 1.89 |  |
| Lymphocyte (10^9) | 1.85 | 0.62 | 1.87 | 0.59 |  |
| Monocyte (10^9) | 0.52 | 0.22 | 0.58 | 0.88 |  |
| Eosinophil (10^9) | 0.16 | 0.21 | 0.13 | 0.16 |  |
| Basophilic (10^9) | 0.07 | 0.40 | 0.08 | 0.26 |  |
| PLT (10^9) | 231.40 | 69.98 | 227.57 | 76.69 |  |
| PT (s) | 11.29 | 0.97 | 10.41 | 0.83 |  |
| INR | 0.98 | 0.07 | 0.88 | 0.08 |  |
| APTT (s) | 32.44 | 4.03 | 24.48 | 4.08 |  |
| Fibrinogen (g/L) | 3.91 | 1.37 | 3.63 | 1.10 |  |
| Thrombin time (s) | 16.40 | 1.08 | 17.67 | 2.88 |  |
| Cholesterol (mmol/L) | 5.00 | 1.07 | 4.93 | 1.74 |  |
| Triglyceride (mmol/L) | 1.40 | 1.00 | 1.54 | 3.09 |  |
| HDL (mmol/L) | 1.35 | 0.77 | 1.93 | 6.31 |  |
| LDL (mmol/L) | 2.86 | 0.73 | 3.00 | 3.60 |  |
| Glucose (mmol/L) | 5.57 | 0.91 | 5.98 | 2.05 |  |
| AST (U/L) | 19.92 | 7.91 | 22.62 | 23.40 |  |
| ALT (U/L) | 20.94 | 14.60 | 26.47 | 39.71 |  |
| Alkaline phosphatase (U/L) | 75.35 | 23.94 | 78.22 | 20.59 |  |
| Total protein (g/L) | 72.10 | 7.91 | 72.20 | 5.52 |  |
| Albumin (g/L) | 42.90 | 4.24 | 44.04 | 3.76 |  |
| Globulin (g/L) | 29.84 | 4.27 | 27.68 | 5.58 |  |
| A/G | 1.47 | 0.24 | 1.62 | 0.30 |  |
| Lactate dehydrogenase (U/L) | 174.55 | 38.57 | 176.74 | 39.06 |  |
| Rate of CO2 (mmol/L) | 22.78 | 2.94 | 25.19 | 3.40 |  |
| K (mmol/L) | 4.12 | 0.33 | 4.28 | 0.33 |  |
| Na (mmol/L) | 143.26 | 3.59 | 139.15 | 3.25 |  |
| Ca (mmol/L) | 2.33 | 0.13 | 2.27 | 0.16 |  |
| Uric acid (umol/L) | 297.79 | 91.06 | 270.96 | 74.03 |  |
| Creatinine (umol/L) | 69.02 | 14.60 | 65.88 | 23.84 |  |
| BUN (umol/L) | 5.70 | 1.32 | 6.42 | 6.71 |  |

**Supplement Table 1. The mean values with standard deviation (SD) for all measurement data**
