# Supplementary material for: A nomogram to predict outcomes of lung cancer patients after pneumonectomy based on 47 indicators
Source: Cancer Med. 2020 Jan 3;9(4):1430–40. doi: 10.1002/cam4.2805 (PMC7013057; doi:10.1002/cam4.2805)
Supplement: Supplementary file 2 [file CAM4-9-1430-s002.docx]

**Supplement Table 3. The component score coefficient matrix**

|  | PC1 | PC2 | TC1 | TC2 | Tc3 | TC4 | BE1 | BE2 | CF1 | CF2 | GM1 | GM2 | LF1 | LF2 | LF3 | Bio1 | Bio2 | RF |
| --- | --- | --- | --- | --- | --- | --- | --- | --- | --- | --- | --- | --- | --- | --- | --- | --- | --- | --- |
| **Gender** | -0.362 | 0.041 |  |  |  |  |  |  |  |  |  |  |  |  |  |  |  |  |
| **Age** | 0.264 | 0.328 |  |  |  |  |  |  |  |  |  |  |  |  |  |  |  |  |
| **Smoke** | 0.384 | -0.203 |  |  |  |  |  |  |  |  |  |  |  |  |  |  |  |  |
| **Drink** | 0.313 | -0.301 |  |  |  |  |  |  |  |  |  |  |  |  |  |  |  |  |
| **HBP** | 0.144 | 0.482 |  |  |  |  |  |  |  |  |  |  |  |  |  |  |  |  |
| **Diabetes** | 0.050 | 0.624 |  |  |  |  |  |  |  |  |  |  |  |  |  |  |  |  |
| **Tumor site** |  |  | 0.043 | 0.253 | 0.355 | -0.450 |  |  |  |  |  |  |  |  |  |  |  |  |
| **Tumor size** |  |  | 0.358 | -0.158 | 0.313 | -0.110 |  |  |  |  |  |  |  |  |  |  |  |  |
| **LNR** |  |  | 0.237 | 0.358 | -0.242 | 0.328 |  |  |  |  |  |  |  |  |  |  |  |  |
| **T stage** |  |  | 0.376 | -0.121 | 0.288 | -0.050 |  |  |  |  |  |  |  |  |  |  |  |  |
| **N stage** |  |  | 0.280 | 0.353 | -0.204 | 0.147 |  |  |  |  |  |  |  |  |  |  |  |  |
| **Pathology** |  |  | -0.130 | 0.355 | 0.379 | 0.340 |  |  |  |  |  |  |  |  |  |  |  |  |
| **Differentiation** |  |  | -0.183 | 0.301 | 0.314 | -0.170 |  |  |  |  |  |  |  |  |  |  |  |  |
| **Treatment** |  |  | -0.041 | -0.215 | 0.352 | 0.689 |  |  |  |  |  |  |  |  |  |  |  |  |
| **Hb** |  |  |  |  |  |  | 0.132 | 0.355 |  |  |  |  |  |  |  |  |  |  |
| **RBC** |  |  |  |  |  |  | 0.246 | 0.257 |  |  |  |  |  |  |  |  |  |  |
| **Neutrophil** |  |  |  |  |  |  | 0.425 | -0.355 |  |  |  |  |  |  |  |  |  |  |
| **Lymphocyte** |  |  |  |  |  |  | 0.184 | 0.430 |  |  |  |  |  |  |  |  |  |  |
| **Monocyte** |  |  |  |  |  |  | 0.442 | -0.297 |  |  |  |  |  |  |  |  |  |  |
| **Eosinophil** |  |  |  |  |  |  | 0.220 | 0.406 |  |  |  |  |  |  |  |  |  |  |
| **Basophilic** |  |  |  |  |  |  | -0.067 | -0.058 |  |  |  |  |  |  |  |  |  |  |
| **PLT** |  |  |  |  |  |  |  |  | 0.181 | 0.400 |  |  |  |  |  |  |  |  |
| **PT** |  |  |  |  |  |  |  |  | 0.330 | -0.044 |  |  |  |  |  |  |  |  |
| **APTT** |  |  |  |  |  |  |  |  | 0.303 | -0.252 |  |  |  |  |  |  |  |  |
| **Fibrinogen** |  |  |  |  |  |  |  |  | 0.215 | 0.484 |  |  |  |  |  |  |  |  |
| **TT** |  |  |  |  |  |  |  |  | 0.165 | -0.494 |  |  |  |  |  |  |  |  |
| **LNR** |  |  |  |  |  |  |  |  | 0.320 | -0.014 |  |  |  |  |  |  |  |  |
| **Cholesterol** |  |  |  |  |  |  |  |  |  |  | 0.488 | -0.001 |  |  |  |  |  |  |
| **Triglyceride** |  |  |  |  |  |  |  |  |  |  | 0.200 | 0.534 |  |  |  |  |  |  |
| **HDL** |  |  |  |  |  |  |  |  |  |  | 0.171 | -0.525 |  |  |  |  |  |  |
| **LDL** |  |  |  |  |  |  |  |  |  |  | 0.471 | -0.034 |  |  |  |  |  |  |
|  | PC1 | PC2 | TC1 | TC2 | Tc3 | TC4 | BE1 | BE2 | CF1 | CF2 | GM1 | GM2 | LF1 | LF2 | LF3 | Bio1 | Bio2 | RF |
| **Glucose** |  |  |  |  |  |  |  |  |  |  | -0.001 | 0.535 |  |  |  |  |  |  |
| **AST** |  |  |  |  |  |  |  |  |  |  |  |  | 0.085 | 0.374 | -0.337 |  |  |  |
| **ALT** |  |  |  |  |  |  |  |  |  |  |  |  | 0.130 | 0.324 | -0.436 |  |  |  |
| **ALP** |  |  |  |  |  |  |  |  |  |  |  |  | -0.175 | 0.124 | -0.184 |  |  |  |
| **Total protein** |  |  |  |  |  |  |  |  |  |  |  |  | -0.120 | 0.330 | 0.431 |  |  |  |
| **Albumin** |  |  |  |  |  |  |  |  |  |  |  |  | 0.220 | 0.280 | 0.432 |  |  |  |
| **Globulin** |  |  |  |  |  |  |  |  |  |  |  |  | -0.384 | 0.195 | 0.138 |  |  |  |
| **A/G** |  |  |  |  |  |  |  |  |  |  |  |  | 0.435 | 0.001 | 0.143 |  |  |  |
| **LDH** |  |  |  |  |  |  |  |  |  |  |  |  |  |  |  | 0.238 | -0.483 |  |
| **Rate of CO2** |  |  |  |  |  |  |  |  |  |  |  |  |  |  |  | -0.094 | 0.743 |  |
| **K** |  |  |  |  |  |  |  |  |  |  |  |  |  |  |  | 0.413 | 0.031 |  |
| **Na** |  |  |  |  |  |  |  |  |  |  |  |  |  |  |  | 0.460 | 0.376 |  |
| **Ca** |  |  |  |  |  |  |  |  |  |  |  |  |  |  |  | 0.570 | -0.001 |  |
| **Uric acid** |  |  |  |  |  |  |  |  |  |  |  |  |  |  |  |  |  | 0.430 |
| **Creatinine** |  |  |  |  |  |  |  |  |  |  |  |  |  |  |  |  |  | 0.469 |
| **BUN** |  |  |  |  |  |  |  |  |  |  |  |  |  |  |  |  |  | 0.354 |
