# Supplementary material for: A nomogram to predict outcomes of lung cancer patients after pneumonectomy based on 47 indicators
Source: Cancer Med. 2020 Jan 3;9(4):1430–40. doi: 10.1002/cam4.2805 (PMC7013057; doi:10.1002/cam4.2805)
Supplement: Supplementary file 3 [file CAM4-9-1430-s003.docx]

| **Category name (Abbreviation)** | **Clinical indicators** | **Principle components** | **Cut-off values** |
| --- | --- | --- | --- |
| Patient characteristic (PC) | Gender, age, smoke habit, drink habit, HBP, diabetes | PC1 | -0.74 |
|  |  | PC2 | -0.44 |
| Tumor characteristic (TC) | Tumor site, size, LNR, T stage, N stage, Pathology type, differentiation, treatment | TC1 | -0.08 |
|  |  | TC2 | 0.63 |
|  |  | TC3 | 0.11 |
|  |  | TC4 | -0.23 |
| Blood routine examination (BE) | Hb, RBC, Neutrophil, Lymphocyte, Monocyte, Eosinophil, Basophilic | BE1 | 0.64 |
|  |  | BE2 | 0.28 |
| Coagulation function (CF) | PLT, PT, INR, APTT, Fibrinogen, Thrombin time | CF1 | 0.27 |
|  |  | CF2 | 1.01 |
| Glucolipid metabolism (GM) | Cholesterol, Triglyceride, HDL, LDL, Glucose | GM1 | 0.15 |
|  |  | GM2 | -0.38 |
| Liver function (LF) | AST, ALT, ALP, Total protein, Albumin, Globulin, A/G | LF1 | 0.0045 |
|  |  | LF2 | 0.70 |
|  |  | LF3 | -0.02 |
| Biochemical and electrolyte (Bio) | K, Na, Ca, Lactate dehydrogenase, Rate of CO_2_ | Bio1 | -0.78 |
|  |  | Bio2 | 0.22 |
| Renal function (RF) | Uric acid, Creatinine, BUN | RF | 0.05 |

**Supplement Table 2. Principle components extraction and cut-off values**

Abbreviations: HBP, high blood pressure; LNR, lymph nodes ratio; Hb, haemoglobin; RBC, red blood cell; PLT, platelet; PT, Prothrombin Time; INR, International Normalized Ratio; APTT, Activated Partial Thromboplastin Time; HDL, High Density Lipoprotein; LDL, Low Density Lipoprotein; AST, glutamic-oxalacetic transaminase; ALT, glutamic-pyruvic transaminase; ALP, alkaline phosphatase; A/G, Albumin-to-Globulin ratio; BUN, Blood Urea Nitrogen.
