# Supplementary material for: A nomogram to predict outcomes of lung cancer patients after pneumonectomy based on 47 indicators
Source: Cancer Med. 2020 Jan 3;9(4):1430–40. doi: 10.1002/cam4.2805 (PMC7013057; doi:10.1002/cam4.2805)
Supplement: Supplementary file 4 [file CAM4-9-1430-s004.docx]

**Formula for each principle:**

PC1=SQRT(2.129)*(-0.362*ZGenda+0.264*ZAge+0.384*ZSmoke+0.313*ZDrink+0.144*ZHBP+0.05*ZDiabate)

PC2=SQRT(1.159)*(0.041*ZGenda+0.328*Zagedata-0.203*ZSmoke-0.301*ZDrink+0.482*ZHBP+0.624*ZDiabate)

TC1=SQRT(2.183)*(0.043*ZTumorsite+0.358*ZTumorsize+0.237*ZLymphonode+0.376*ZT+0.280*ZN-0.130*ZPathology-0.183*ZDifferentiation-0.041*ZChemraiotherapy)

TC2=SQRT(1.614)*(0.253*ZTumorsite-0.158*ZTumorsize+0.358*ZLymphonode-0.121*ZT+0.353*ZN+0.355*ZPathology+0.301*ZDifferentiation-0.215*ZChemraiotherapy)

TC3=SQRT(1.294)*(0.355*ZTumorsite+0.313*ZTumorsize-0.242*ZLymphonode+0.288*ZT-0.204*ZN+0.379*ZPathology+0.314*ZDifferentiation+0.352*ZChemraiotherapy)

TC4= SQRT(1.035)*( -0.450*ZTumorsite-0.11*ZTumorsize+0.328*ZLymphonode-0.050*ZT+0.147*ZN+0.340*ZPathology-0.17*ZDifferentiation+0.689*ZChemraiotherapy)

BE1=SQRT(1.847)*(0.132*ZHb+0.246*ZRBC+0.425*ZNeutrophildata+0.184*ZLymphocyte+0.442*ZMonocyte+0.220*ZEosinophil-0.067*Zbasophilic)

BE2= SQRT(1.316)*(0.355*ZHb+0.257*ZRBC-0.355*ZNeutrophil+0.43*ZLymphocyte-0.297*ZMonocyte+0.406*ZEosinophil-0.058*ZBasophilic)

CF1=SQRT(2.443)*(0.181*ZPLT+0.33*ZPT+0.303*ZAPTT+0.215*Zfabrinogendata+0.165*ZThrombintime+0.32*ZINRdata)

CF2= SQRT(1.421)*(0.4*ZPLT-0.044*ZPT-0.252*ZAPTT+0.484*Zfabrinogendata-0.494*ZThrombintime-0.014*ZINRdata)

GM1=SQRT(1.888)*(0.488*Zcholesterol+0.2*Ztriglyceride+0.171*ZHDL+0.471*ZLDL-0.001*ZGlucose)

GM2=SQRT(1.179)*(-0.001*Zcholesterol+0.534*Ztriglyceride-0.525*ZHDL-0.034*ZLDL+0.535*ZGlucose)

LF1=SQRT(2.198)*(0.085*ZAST+0.13*ZALT-0.175*ZAlkalinephosphata-0.12*Ztotalprotein+0.22*Zalbumin-0.384*Zglobulin+0.435*ZAGratio)

LF2= SQRT(2.061)*(0.374*ZAST+0.324*ZALT+0.124*ZAlkalinephosphata +0.33*Ztotalprotein+0.28*Zalbumin+0.195*Zglobulin+0.001*ZAGratio)

LF3= SQRT(1.334)*(-0.337*ZAST-0.436*ZALT-0.184*ZAlkalinephosphata+0.431*Ztotalprotein+0.432*Zalbumin+0.138*Zglobulin+0.143*ZAGratio)

Bio1=SQRT(1.295)*(0.238*ZLactatedehydrogenasedata-0.094*ZrateofCO2+0.413*ZK+0.46*ZNa+0.57*ZCa)

Bio2=SQRT(1.077)*(-0.483*ZLactatedehydrogenasedata+0.743*ZrateofCO2+0.031*ZK+0.376*ZNa-0.001*ZCa)

Bio1=SQRT(1.295)*(0.238*ZLactatede hydrogenasedata-0.094*Zrate of CO2+0.413*ZK+0.46*ZNa+0.57*ZCa)

Bio2=SQRT(1.077)*(-0.483*ZLactatede hydrogenasedata+0.743*Zrate of CO2+0.031*ZK+0.376*ZNa-0.001*ZCa)

RF1=SQRT(1.884)*(0.43*ZUric acid+0.469*Zcreatinine+0.354*ZBUN)

*Zx: standardization value of x.

SQRT, Square
